# Serum Glycated Albumin Is Inversely Influenced by Fat Mass and Visceral Adipose Tissue in Chinese with Normal Glucose Tolerance

**DOI:** 10.1371/journal.pone.0051098

**Published:** 2012-11-29

**Authors:** Feifei Wang, Xiaojing Ma, Yaping Hao, Rong Yang, Jie Ni, Yunfeng Xiao, Junling Tang, Yuqian Bao, Weiping Jia

**Affiliations:** 1 Department of Endocrinology and Metabolism, Shanghai Jiao Tong University Affiliated Sixth People’s Hospital, Shanghai Clinical Center for Diabetes, Shanghai Diabetes Institute, Shanghai Key Laboratory of Diabetes Mellitus, Shanghai, China; 2 Department of Radiology, Shanghai Jiao Tong University Affiliated Sixth People’s Hospital, Shanghai, China; University of Hong Kong, China

## Abstract

**Background:**

Recent studies have revealed that body mass index (BMI) inversely influenced serum glycated albumin (GA), which may cause an underestimation of GA-monitored short-term hyperglycemic control.

**Objective:**

This study was to investigate the association between anthropometric variables (BMI and waist circumference (W)) and accurate adiposity variables (percentage of body fat (%fat), fat mass, free fat mass (FFM), subcutaneous fat area (SFA), and visceral fat area (VFA)) with serum GA.

**Design:**

A total of 2563 subjects (1037 men, 593 premenopausal women, and 933 postmenopausal women) with normal glucose tolerance underwent bioelectrical impedance body fat content measurement and magnetic resonance imaging. Serum GA and absolute value of GA (aGA) were measured by enzymatic assay.

**Results:**

Compared to the BMI <25.0 kg/m^2^ group, the BMI ≥25.0 kg/m^2^ group had significantly higher fasting plasma glucose, glycated hemoglobin A1c, and body fat parameters including W, %fat, fat mass, FFM, SFA, and VFA, but significantly lower aGA, and GA in all the three sex- and menopause-stratified groups (all *P*<0.05). GA decreased with the increment of fat mass for all three groups (all *P* for trend <0.001). In the same BMI category, men and postmenopausal women with elevated %fat (men, ≥25%; women, ≥35%) still had significantly lower GA than those with normal %fat (men, <25%; women, <35%) (all *P*<0.05). Multiple stepwise regression showed that %fat, fat mass, and VFA were independently associated with GA.

**Conclusions:**

Serum GA was inversely influenced by fat mass and visceral adipose tissue in Chinese with normal glucose tolerance.

## Introduction

Serum glycated albumin (GA) has emerged as a popular and useful clinical measurement of glycemic control in diabetic patients [1]. However, as GA is influenced by the half-life of serum albumin (ALB), it may underestimate the actual plasma glucose concentration under conditions of vigorous ALB turnover [2]. Disorders such as hyperthyroidism, hypothyroidism, nephrotic syndrome, and liver cirrhosis are known to affect GA [3].

Recently, studies have suggested a negative correlation between obesity and serum GA [4]–[7]. Serum GA was found to be relatively low in both obese non-diabetic children [4] and obese diabetic adult patients [5], as compared with non-obese matched subjects. Two other studies demonstrated a significantly inverse correlation between body mass index (BMI) and serum GA in adults, regardless of diabetes status [6], [7].

As an anthropometric index, BMI is widely used in clinic setting to assess obesity status. However, BMI is unable to distinguish between fat mass and free fat mass (FFM), thus providing an inadequate estimate of body fat content [8]–[10]. It has been demonstrated that body fat, rather than body weight, is associated closely with increased risks of obesity-related disorders [9], [11], [12]. Moreover, elevated content of abdominal adipose tissue, especially visceral adipose tissue, represents a significantly high risk of obesity-related complications [13], [14].

Waist circumference (W) is a simple anthropometric index for abdominal obesity, while subcutaneous fat area (SFA) and visceral fat area (VFA) are frequently-used accurate variables. Therefore, this study was designed to investigate whether fat mass or FFM acts as the primary factor negatively influencing serum GA, and to determine the effects of common abdominal obesity variables, including W, SFA, and VFA, on serum GA.

To our knowledge, no study in the literature to date has focused on the relationship between accurate adiposity variables and serum GA in a Chinese population. In the present study, we examined the association between body fat, as well as visceral adiposity, and serum GA in a Chinese population. Only individuals with normal glucose tolerance (NGT) were enrolled, in order to avoid the influence of hyperglycemia (impaired glucose regulation (IGR) or diabetes) on GA [7], [15].

**Table 1:** Clinical and biochemical characteristics of the study subjects.

Parameters	Men	Premenopausal women	Postmenopausal women
N	1037	593	933
Age (years)	52.8±9.0	44.2±6.6**	56.3±4.6** ††
BMI (kg/m^2^)	23.9 (21.9–25.8)	22.4 (20.5–24.6)**	22.8 (20.9–24.7)**
W (cm)	85.0 (79.0–91.0)	76.0 (70.3–81.5)**	78.0 (73.0–84.0)** ††
%fat	22.5±5.5	30.2±6.2**	30.2±6.1**
Fat mass (kg)	15.6 (11.8–19.3)	16.9 (13.6–21.2)**	17.3 (13.7–21.3)**
FFM (kg)	53.0 (49.3–57.4)	39.7 (37.5–42.2)**	40.0 (37.6–42.3)**
SFA (cm^2^)	136.2 (104.5–175.6)	167.1 (134.6–208.0)**	200.3 (161.4–242.4)** ††
VFA (cm^2^)	88.8 (60.1–121.0)	52.4 (37.6–74.7)**	70.4 (52.6–90.7)** ††
FPG (mmol/L)	5.23±0.42	5.14±0.42**	5.17±0.39**
2hPG (mmol/L )	5.7 (4.8–6.6)	5.7 (5.0–6.5)	6.2 (5.5–6.9)** ††
HbA1c (%)	5.5 (5.3–5.7)	5.4 (5.2–5.7)**	5.6 (5.4–5.8)** ††
GA (%)	13.57±1.22	13.98±1.28**	14.14±1.13** †
ALB (g/dL )	4.72±0.33	4.68±0.33**	4.70±0.30
aGA (g/dL )	0.57±0.07	0.59±0.08**	0.60±0.07** ††
SBP (mmHg)	122.7 (116.0–131.3)	119.3 (107.3–125.0)**	120.0 (110.3–130.0)** ††
DBP (mmHg)	79.3 (73.3–84.3)	77.3 (70.0–80.0)**	76.7 (70.0–80.7)**
TC (mmol/L)	4.9 (4.3–5.5)	4.7 (4.2–5.3)**	5.5 (4.8–6.1)** ††
TG (mmol/L)	1.4 (1.0–1.9)	0.9 (0.7–1.4)**	1.2 (0.9–1.7)** ††
HDL-c (mmol/L)	1.3 (1.1–1.5)	1.5 (1.3–1.8)**	1.6 (1.3–1.8)**
LDL-c (mmol/L	3.2 (2.7–3.8)	3.0 (2.4–3.5)**	3.4 (2.9–4.0)** ††
CRP (mg/L)	0.7 (0.3–1.3)	0.4 (0.2–0.9)**	0.7 (0.3–1.3)††
Current smoker, N (%)	603 (58.1%)	15 (2.5%)**	14 (1.5%)**
Hypertension, N (%)	299 (28.8%)	72 (12.1%)**	237 (25.4%)††
Anti-hypertensive therapy, N (%)	143 (13.8%)	42 (7.1%)**	151 (16.2%)††
Hypertriglyceridemia, N (%)	335 (32.3%)	86 (14.5%)**	223 (23.9%)** ††
Hypercholesterolemia, N (%)	378 (36.5%)	180 (30.4%)*	590 (63.2)** ††
Lipid-lowering therapy, N (%)	8 (0.8%)	2 (0.3%)	24 (2.6%)** ††
Low HDL-c, N (%)	177 (17.1%)	21 (3.5%)**	22 (2.4%)**

* *P*<0.05

** *P*<0.01 versus men;

† *P*<0.05

†† *P*<0.01 versus premenopausal women.

Abbreviations: ALB, albumin; aGA, absolute value of GA; CRP, C-reactive protein; DBP, diastolic blood pressure; FFM, free fat mass; FPG, fast plasma glucose; GA, glycated albumin; HDL-c, high-density lipoprotein cholesterol; LDL-c, low-density lipoprotein cholesterol; SFA, subcutaneous fat area; SBP, Systolic blood pressure; TG, triglyceride; TC, total cholesterol; VFA, visceral fat area; W, waist circumference; 2hPG, 2-h post-OGTT plasma glucose. Data are mean ± SD or median (interquartile range).

## Methods

### Ethics Statement

The study was approved by the Ethics Committee of Shanghai Jiao Tong University affiliated Sixth People’s Hospital, and all subjects provided written informed consent prior to study participation.

**Table 2:** Clinical and biochemical characteristics of the study subjects by BMI category.

Parameters	Men			Premenopausal women		Postmenopausal women	
	BMI<25 kg/m^2^	BMI≥25 kg/m^2^		BMI<25 kg/m^2^	BMI≥25 kg/m^2^	BMI<25 kg/m^2^	BMI≥25 kg/m^2^
N	689		348	462	131	726	207
Age (years)	53.2±8.8		52.1±9.4	43.5±6.8	46.4±5.7§§	56.3±4.5	56.1±4.9
BMI (kg/m^2^)	22.7 (20.9–23.9)		26.7 (25.8–28.2)‡‡	21.6 (20.2–23.0)	26.8 (25.8–28.4)§§	22.1 (20.4–23.4)	26.7 (25.6–28.0)
W (cm)	81.0 (76.5–86.0)		93.5 (89.0–98.5) ‡‡	73.0 (69.5–78.0)	86.0 (81.0–90.0)§§	76.0 (72.0–80.5)	88.0 (84.0–92.0)
%fat	20.3±4.4		27.0±4.7‡‡	27.9±4.4	38.4±4.7§§	28.1±4.8	37.3±4.4
Fat mass (kg)	13.2 (10.5–16.0)		21.0 (17.7–25.3) ‡‡	15.5 (12.9–18.0)	25.5 (23.2–29.9)§§	15.7 (12.7–18.6)	25.1 (22.4–27.9)
FFM (kg)	51.1 (47.9–54.2)		58.1 (54.5–61.5) ‡‡	39.1 (37.1–41.4)	42.2 (40.0–45.4)§§	39.5 (37.2–41.4)	42.3 (40.1–44.9)
SFA (cm^2^)	116.9 (92.3–144.8)		181.0 (152.6–220.4) ‡‡	156.3 (127.1–187.2)	232.4 (193.3–276.3)§§	186.1 (151.4–217.7)	259.5 (218.8–303.4)
VFA (cm^2^)	72.1 (48.5–99.2)		125.7 (95.8–153.5) ‡‡	47.4 (34.5–62.3)	82.8 (66.0–106.2)§§	63.5 (49.2–81.2)	97.4 (83.7–119.1)
FPG (mmol/L)	5.2±0.4		5.3±0.4‡‡	5.1±0.4	5.2±0.4§§	5.1±0.4	5.3±0.4
2hPG (mmol/L )	5.7 (4.8–6.5)		5.8 (4.7–6.8)	5.7 (5.0–6.5)	5.8 (5.0–6.6)	6.1 (5.4–6.8)	6.6 (5.8–7.2)
HbA1c (%)	5.5 (5.3–5.7)		5.6 (5.3–5.8) ‡‡	5.4 (5.2–5.6)	5.5 (5.3–5.7)§	5.6 (5.4–5.8)	5.7 (5.5–5.9)
GA (%)	13.70±1.21		13.33±1.19‡‡	14.13±1.26	13.45±1.25§§	14.28±1.07	13.62±1.18
ALB (g/dL)	4.73±0.34		4.72±0.33	4.70±0.33	4.59±0.33§§	4.71±0.30	4.66±0.31
aGA (g/dL)	0.58±0.07		0.56±0.06‡‡	0.60±0.07	0.55±0.07 §§	0.61±0.07	0.57±0.07
SBP (mmHg)	120.7 (113.0 –130.0)		127.0 (120.0–139.2) ‡‡	116.7 (105.3–123.3)	121.3 (116.3–130.0)§§	120.0 (110.0–129.3)	123.3 (113.3–131.3)
DBP (mmHg)	79.3 (71.3–82.0)		80.0 (76.7–88.7) ‡‡	76.0 (70.0–80.0)	79.3 (72.7–81.3)§§	76.0 (70.0–80.0)	79.0 (69.3–82.7)
TC (mmol/L )	4.9 (4.4–5.5)		4.9 (4.3–5.5)	4.7 (4.2–5.3)	4.9 (4.3–5.4)	5.5 (4.8–6.1)	5.5 (5.0–6.1)
TG (mmol/L )	1.3 (0.9–1.8)		1.5 (1.1–2.2) ‡‡	0.9 (0.7–1.3)	1.2 (0.9–1.7)§§	1.1 (0.8–1.6)	1.4 (1.1–1.9)
HDL-c (mmol/L)	1.3 (1.1–1.6)		1.2 (1.0–1.3) ‡‡	1.5 (1.3–1.8)	1.5 (1.3–1.6)§§	1.6 (1.4–1.8)	1.4 (1.2–1.7)
LDL-c (mmol/L)	3.2 (2.7–3.7)		3.3 (2.7–3.9) ‡	2.9 (2.4–3.4)	3.2 (2.7–3.8)§§	3.3 (2.8–3.9)	3.5 (3.1–4.0)
CRP (mg/L )	0.6 (0.3–1.1)		0.9 (0.5–1.9) ‡‡	0.3 (0.2–0.7)	0.8 (0.4–1.5)§§	0.5 (0.3–1.0)	1.2 (0.7–2.4)
Current smoker, N (%)	404 (58.6%)		199 (57.2%)	13 (2.8%)	2 (1.5%)§	10 (1.4%)	4 (1.9%)
Hypertension, N (%)	160 (23.2%)		139 (39.9%)‡‡	39 (8.4%)	33 (25.2%)§§	149 (20.5%)	88 (42.5%)
Anti-hypertensive therapy, N (%)	78 (11.3%)		65 (18.7%)‡‡	19 (4.1%)	23 (17.6%)§§	91 (12.5%)	60 (29.0%)
Hypertriglyceridemia, N (%)	191 (27.7%)		144 (41.4%)‡‡	55 (11.9%)	31 (23.7%)§§	146 (20.1%)	77 (37.2%)
Hypercholesterolemia, N (%)	254 (36.9%)		124 (35.6%)	134 (29.0%)	46 (35.1%)	448 (61.7%)	142 (68.6%)
Lipid-loweringtherapy, N (%)	4 (0.6%)		4 (1.1%)	2 (0.4%)	0 (0.0%)	15 (2.1%)	9 (4.3%)
Low HDL-c, N (%)	96 (13.9%)		81 (23.3%)‡‡	13 (2.8%)	8 (6.1%)	14 (1.9%)	8 (3.9%)

‡ *P*<0.05

‡‡ *P*<0.01 versus men with BMI<25k****g/m^2^;

§ *P*<0.05

§§ *P*<0.01 versus premenopausal women with BMI<25 kg/m^2^;

¶ *P*<0.05

¶¶ *P*<0.01 versus postmenopausal women with BMI<25 kg/m^2^.

Abbreviations: ALB, albumin; aGA, absolute value of GA; CRP, C-reactive protein; DBP, diastolic blood pressure; FFM, free fat mass; FPG, fast plasma glucose; GA, glycated albumin; HDL-c, high-density lipoprotein cholesterol; LDL-c, low-density lipoprotein cholesterol; SFA, subcutaneous fat area; SBP, Systolic blood pressure; TG, triglyceride; TC, total cholesterol; VFA, visceral fat area; W, waist circumference; 2hPG, 2-h post-OGTT plasma glucose. Data are mean ± SD or median (interquartile range).

### Study Population

MRI examination was performed in 3066 subjects (≥20 years old) with NGT from four communities in Shanghai (Baoshan, Gonghexin, Tianmuxi and Daning commuties, respectively) from December 2009 to December 2011. Each subject was invited to complete a questionnaire about present and past illness, as well as current and previous medical therapy. All subjects had complete anthropometric indices and lab data. A 75 g oral glucose tolerance test (OGTT) was administered to each subject and glucose tolerance status was diagnosed according to the 1999 WHO criteria [16].

**Figure 1:**
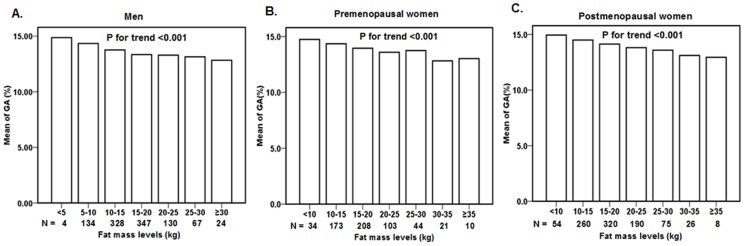
Serum GA of subjects with different fat mass levels. The men, premenopausal women, and postmenopausal women groups were further stratified among seven subgroups of fat mass level respectively, according to 5 kg increments of fat mass. This stratification revealed that GA decreased as fat mass increased for all three groups (all *P* for trend <0.001).

The exclusion criteria included the following: 1) chronic hepatic disease or hypoproteinemia (n = 45); 2) chronic kidney disease or undergoing hemodialysis (n = 16); 3) hyperthyroidism or hypothyroidism (n = 151); 4) presence of cancer (n = 21); 5) hematological abnormalities or anemia (n = 51); 6) history of cardiovascular disease (n = 80); 7) psychiatric disturbance or blind person (n = 9); 8) current treatment with systemic corticosteroids (n = 6); 9) current infectious conditions (with increased white blood cell count or urinary tract infection) (n = 76); 10) C-reactive protein (CRP) levels >10****mg/L (n = 48). Finally, a total of 2563 NGT subjects (age range: 21–75 years old) were included in this study.

**Figure 2:**
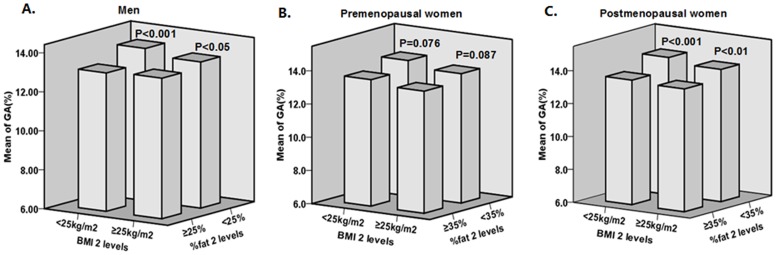
Serum GA of subjects with different %fat levels in the same BMI category. In the same BMI category, subjects with elevated %fat (≥25% for men, ≥35% for women) had significantly lower GA than those with normal %fat (<25% for men, <35% for women) in both men and postmenopausal women (all *P*<0.05), while in premenopausal women, the differences were not significant (*P* = 0.076 and *P* = 0.087 respectively).

**Table 3:** Correlation and partial correlation with serum GA.

Parameters	Men				Premenopausal women				Postmenopausal women			
	r	*P*	r	*P*	r	*P*	r	*P*	r	*P*	r	*P*
			(Adjusted△)				(Adjusted△)				(Adjusted△)	
age	0.187	<0.001	–	–	0.010	0.799	–	–	0.081	0.013	–	–
BMI	−0.256	<0.001	–	–	−0.303	<0.001	–	–	−0.342	<0.001	–	–
W	−0.252	<0.001	−0.094	0.003	−0.279	<0.001	−0.070	0.042	−0.343	<0.001	−0.111	<0.001
%fat	−0.310	<0.001	−0.154	<0.001	−0.302	<0.001	−0.090	0.030	−0.391	<0.001	−0.195	<0.001
Fat mass	−0. 311	<0.001	−0.169	<0.001	−0.307	<0.001	−0.098	0.018	−0.379	<0.001	−0.135	<0.001
FFM	−0.141	<0.001	0.066	0.034	−0.128	0.002	−0.003	0.944	−0.083	0.011	0.132	0.058
SFA	−0.206	<0.001	−0.020	0.021	−0.294	<0.001	−0.129	0.002	−0.271	<0.001	−0.084	0.010
VFA	−0.281	<0.001	−0.153	<0.001	−0.310	<0.001	−0.169	<0.001	−0.366	<0.001	−0.212	<0.001
FPG	0.114	<0.001	0.152	<0.001	0.147	<0.001	0.223	<0.001	0.098	0.003	0.170	<0.001
2hPG	0.050	0.105	0.046	0.143	0.011	0.790	0.054	0.191	0.016	0.623	0.103	0.002
HbA1c	0.120	<0.001	0.110	<0.001	0.023	0.048	0.033	0.431	0.130	<0.001	0.172	<0.001
ALB	−0.220	<0.001	–	–	−0.080	0.047	–	–	−0.118	<0.001	–	–
SBP	–0.105	0.002	–0.082	0.008	–0.018	0.665	0.086	0.038	–0.052	0.115	–0.007	0.979
DBP	–0.103	0.002	–0.045	0.147	–0.034	0.415	0.047	0.254	–0.046	0.157	–0.024	0.471
TC	–0.098	0.001	–0.083	0.008	–0.068	0.098	–0.064	0.110	–0.086	0.009	–0.065	0.047
TG	–0.244	<0.001	–0.169	<0.001	–0.262	<0.001	–0.186	<0.001	–0.247	<0.001	–0.144	<0.001
HDL-c	0.135	<0.001	0.036	0.250	0.177	<0.001	0.121	0.003	0.214	<0.001	0.130	<0.001
LDL-c	–0.103	0.001	–0.028	0.378	–0.082	0.046	–0.049	0.240	–0.117	<0.001	–0.081	0.013
CRP	–0.131	<0.001	–0.092	0.003	–0.148	<0.001	–0.060	0.149	–0.137	<0.001	0.012	0.713

△ Adjusted for age, ALB, BMI, smoking, anti-hypertensive therapy, and lipid-lowering therapy.

Abbreviation: ALB, albumin; aGA, absolute value of GA; CRP, C-reactive protein; DBP, diastolic blood pressure; FFM, free fat mass; FPG, fast plasma glucose; GA, glycated albumin; HDL-c, high-density lipoprotein cholesterol; LDL-c, low-density lipoprotein cholesterol; SFA, subcutaneous fat area; SBP, Systolic blood pressure; TG, triglyceride; TC, total cholesterol; VFA, visceral fat area; W, waist circumference; 2hPG, 2-h post-OGTT plasma glucose.

### Anthropometric and Body Fat Measurements

All participants underwent complete physical examination, including measurements of height, weight, W, and blood pressure (BP). BMI (kg/m^2^) was calculated as weight divided by height squared. W was measured on the mid-axillary line between the inferior margin of 12th rib and the iliac crest. Resting BP was determined as the average of three time measures obtained on the right arm using a random-zero sphygmomanometer at an interval of 3 min. Subjects with systolic BP (SBP) ≥140 mmHg and/or diastolic BP (DBP) ≥90 mmHg, or those who were currently receiving treatment for previously diagnosed hypertension, were defined as hypertensive patients. An automatic bioelectrical impedance analyzer (BC-420; Tanita Corp., Tokyo, Japan) was used to estimate body composition, which consists of fat mass and FFM, as well as percentage of body fat (%fat). FFM is the weight of body composition except fat, including muscle, bone, water and other tissue. BMI ≥25.0 kg/m^2^ was defined as overweight/obesity, according to the 1999 WHO criterion [11]. An alternative definition of obesity was used based on %fat (≥25% for men and ≥35% for women), as proposed by the WHO [9], [11].

**Table 4:** Stepwise multivariate regression analyses on serum GA.

Men					Premenopausal women					Postmenopausal women				
Independent Variable	β	SEM	*Standardized β*	*P*	Independent Variable	β	SEM	*Standardized β*	*P*	Independent Variable	β	SEM	*Standardized β*	*P*
Model 1					Model 1					Model 1				
Age	0.013	0.004	0.093	0.002	Age	0.011	0.003	0.052	0.033	Age	0.019	0.007	0.076	0.011
BMI	–0.044	0.021	−0.114	0.035	BMI	−0.125	0.016	−0.308	<0.001	BMI	−0.089	0.019	−0.232	<0.001
W	−0.020	0.007	−0.149	0.007	W	−0.095	0.014	−0.058	0.037	W	−4.010	1.251	−0.159	0.001
FPG	0.472	0.083	0.164	<0.001	FPG	0.724	0.115	0.237	<0.001	FPG	0.522	0.086	0.180	<0.001
2hPG	0.065	0.030	0.062	0.029	2hPG	0.105	0.047	0.084	0.027	2hPG	1.766	0.462	0.117	<0.001
ALB	−0.733	0.109	−0.202	<0.001	ALB	−0.602	0.146	−0.156	<0.001	ALB	−0.555	0.112	−0.148	<0.001
TG	−0.146	0.031	−0.133	<0.001	TG	−0.357	0.081	−0.188	<0.001	TG	−0.880	0.170	−0.164	<0.001
SBP	−0.007	0.003	−0.076	0.010										
Smoking	−0.239	0.038	−0.177	<0.001										
Model 2					Model 2					Model 2				
Age	0.009	0.004	0.067	0.028	Age	0.021	0.008	0.109	0.005	Age	0.019	0.007	0.075	0.011
%fat	−0.046	0.008	−0.206	<0.001	%fat	−0.047	0.010	−0.231	<0.001	%fat	−0.053	0.007	−0.282	<0.001
VFA	−0.003	0.001	−0.121	0.001	VFA	−0.009	0.002	−0.204	<0.001	VFA	−1.206	0.248	−0.198	<0.001
FPG	0.511	0.082	0.177	<0.001	FPG	0.820	0.114	0.269	<0.001	FPG	−1.254	0.454	0.105	<0.001
2hPG	0.066	0.030	0.063	0.026	ALB	−0.465	0.147	−0.120	0.002	2hPG	1.827	0.451	0.121	<0.001
ALB	−0.709	0.107	−0.195	<0.001	TG	−0.316	0.076	−0.166	<0.001	ALB	−0.548	0.109	−0.146	<0.001
TG	−0.129	0.031	−0.118	<0.001						TG	−0.620	0.173	−0.115	<0.001
SBP	−0.006	0.002	−0.074	0.011										
Smoking	−0.224	0.038	−0.166	<0.001										
Model 3					Model 3					Model 3				
Age	0.010	0.004	0.072	0.020	Age	0.021	0.008	0.108	0.006	Age	0.021	0.007	0.084	0.004
Fat mass	−0.042	0.009	−0.204	<0.001	Fat mass	−0.046	0.010	−0.226	<0.001	Fat mass	−2.432	0.342	−0.307	<0.001
VFA	−0.004	0.001	−0.147	0.001	VFA	−0.009	0.002	−0.194	<0.001	VFA	−1.436	0.262	−0.236	<0.001
FPG	0.499	0.082	0.173	<0.001	FPG	0.791	0.114	0.259	<0.001	FPG	0.509	0.084	0.175	<0.001
2hPG	0.068	0.030	0.065	0.023	ALB	−0.463	0.147	−0.120	0.002	2hPG	1.835	0.450	0.122	<0.001
ALB	−0.717	0.108	−0.197	<0.001	TG	−0.328	0.076	−0.172	<0.001	ALB	−0.543	0.108	−0.145	<0.001
TG	−0.130	0.032	−0.118	<0.001						TG	−0.599	0.173	−0.112	0.001
SBP	−0.007	0.003	−0.080	0.007										
Smoking	−0.225	0.038	−0.166	<0.001										

Model 1: Age, BMI, W, FPG, 2hPG, ALB, TG, HDL-c, LDL-c, SBP, DBP, CRP, smoking, anti-hypertensive therapy, lipid-lowering therapy.

Model 2: Age, BMI, W, %fat, SFA, VFA, FPG, 2hPG, ALB, TG, HDL-c, LDL-c, SBP, DBP, CRP, smoking, anti-hypertensive therapy, lipid-lowering therapy.

Model 3: Age, BMI, W, fat mass, FFM, SFA, VFA, FPG, 2hPG, ALB,TG, HDL-c, LDL-c, SBP, DBP, CRP, smoking, anti-hypertensive therapy, lipid-lowering therapy.

Abbreviations: ALB, albumin; aGA, absolute value of GA; CRP, C-reactive protein; DBP, diastolic blood pressure; FFM, free fat mass; FPG, fast plasma glucose; GA, glycated albumin; HDL-c, high-density lipoprotein cholesterol; LDL-c, low-density lipoprotein cholesterol; SFA, subcutaneous fat area; SBP, Systolic blood pressure; TG, triglyceride; TC, total cholesterol; VFA, visceral fat area; W, waist circumference; 2hPG, 2-h post-OGTT plasma glucose.

### MRI

Abdominal adipose tissue, including SFA and VFA, was assessed as previously described [17]. Briefly, a 3.0T clinical MRI scanner (Archiva; Philips Medical System, Amsterdam, The Netherlands) was used to image the abdominal region between the L4 and L5 vertebrae with the subject in the supine position. Segmentation of the images into SFA and VFA was carried out by the Slice-O-Matic image analysis software version 4.2 (Tomovision Inc., Montreal, QC, Canada).

### Laboratory Measurements

After a 10 h overnight fast, blood samples were collected to measure plasma glucose levels and lipid profile. Fasting plasma glucose (FPG) and 2 h post-OGTT plasma glucose (2hPG) were assayed by the glucose oxidase method. ALB, absolute value of GA (aGA) and GA were measured by enzymatic assays using the Hitachi 7600–120 automatic analyzer (Lucica GA-L; Asahi Kasei Pharma Corporation, Tokyo, Japan). Inter-assay and intra-assay coefficients of variation for GA were <5.1% and <3.0%, respectively. Glycated hemoglobin (HbA1c) level was determined by high-pressure liquid chromatography (Bio-Rad Inc., Hercules, CA, USA). Serum triglyceride (TG) and total cholesterol (TC) were measured by enzymatic assays, while high-density lipoprotein cholesterol (HDL-c) and low-density lipoprotein cholesterol (LDL-c) were measured by direct assay method; all using the Hitachi 7600–120 automatic analyzer. The serum concentration of CRP was assayed by particle-enhanced immunonephelometry using the Cardio Phase hs-CRP reagent (Siemens Medical Solutions, Gebaude, Germany). The diagnostic definition for dyslipidemia, including hypertriglyceridemia, hypercholesterolemia, and low HDL-c, followed the 2007 Joint Committee for Developing Chinese Guidelines on Prevention and Treatment of Dyslipidemia (JCDCG 2007) [18].

### Statistical Analysis

All statistical analysis was performed with the Statistical Package for Social Sciences version 16.0 software (SPSS, Chicago, IL, USA). The clinical and biochemical data of the subjects are presented as mean ± SD, except for skewed variables which are presented as median (interquartile range 25–75%). Two-tailed tests and a 5% level of significance were applied in all statistical analyses. Clinical characteristics that followed a normal distribution were compared among the three groups using one-way ANOVA test, and those that were not normally distributed were compared with Kruskal–Wallis test. Intergroup comparisons of variables with normal distribution were carried out by the unpaired Student’s *t-*test, while variables with non-normal distribution were compared by the Wilcoxon rank-sum test. For dichotomous or categorical variables, intergroup comparisons were carried out by the Chi-squared (χ^2^) test. Partial correlation analysis was performed to investigate the association between GA and other parameters adjusted for age, BMI, ALB, smoking, anti-hypertensive therapy, and lipid-lowering therapy. Multiple stepwise regression analysis was used to assess the association of %fat, fat mass, VFA, and other metabolic parameters with GA after adjusting for potential confounders. For all statistical analyses, a *P*-value <0.05 was considered significant.

## Results

### Body Fat Parameters and Clinical Characteristics of Subjects

The final study population included 2563 NGT subjects (1037 men, 593 premenopausal women, and 933 postmenopausal women) aged 52.1±8.5 years. The clinical and biochemical characteristics of the study subjects are displayed in Table 1. Compared with men, both premenopausal and postmenopausal women had significantly lower BMI, W, FFM, VFA, FPG, SBP, DBP, and TG, but higher %fat, fat mass, SFA, and HDL-c (all *P*<0.01). Postmenopausal women had significantly higher age, W, SFA, VFA, 2hPG, HbA1c, GA, aGA, SBP, TC, TG, LDL-c, and CRP than premenopausal women (all *P*<0.05). Moreover, the proportion of current smokers and the frequency of hypertriglyceridemia and low HDL-c were significantly higher in men (all *P*<0.01 vs. women), while the frequency of hypercholesterolemia was significantly higher in postmenopausal women (both *P*<0.01 vs. men and premenopausal women).

To investigate the relationship between body fat parameters and GA levels, we stratified the men, premenopausal women, and postmenopausal women groups into subgroups of non-overweight/non-obese (BMI <25 kg/m^2^) and overweight/obese (BMI ≥25 kg/m^2^) respectively, as shown in Table 2. Compared with the non-overweight/non-obese subjects, body fat parameters (W, %fat, fat mass, FFM, SFA, and VFA) were found to be significantly higher in the overweight/obese subjects (all *P*<0.01). The overweight/obese subjects also had significantly higher FPG, HbA1c, SBP, TG, and CRP, but significantly lower aGA, GA, and HDL-c than the non-overweight/non-obese subjects (all *P*<0.05). ALB was significantly lower in both premenopausal and postmenopausal overweight/obese women, compared to non-overweight/non-obese women (both *P*<0.05). The frequency of hypertension, anti-hypertensive therapy, and hypertriglyceridemia were significantly higher in overweight/obese subjects (all *P*<0.05 vs. non-overweight/non-obese subjects), while the frequency of hypercholesterolemia and lipid-lowering therapy was not significantly different between the two subgroups in all three groups (all *P*>0.05).

### Relationship between Body Fat and Serum GA

The men, premenopausal women, and postmenopausal women groups were further stratified among seven subgroups of fat mass level, according to 5 kg increments of fat mass (Figure 1). This stratification revealed that GA decreased as fat mass increased for all three groups (all *P* for trend <0.001).

To investigate the influence of %fat on GA levels, men, premenopausal women, and postmenopausal women were further stratified among four %fat subgroups, as follows: subgroup 1 with BMI <25 kg/m^2^ and normal %fat (<25% for men, <35% for women); subgroup 2 with BMI <25 kg/m^2^ but elevated %fat (≥25% for men, ≥35% for women); subgroup 3 with BMI ≥25 kg/m^2^ but normal %fat; subgroup 4 with BMI ≥25 kg/m^2^ and elevated %fat. As displayed in Figure 2, in the same BMI category, subjects with elevated %fat still had significantly lower GA than those with normal %fat in both men and postmenopausal women (all *P*<0.05), while in premenopausal women the differences were not significant (*P* = 0.076 and *P* = 0.087, respectively).

### Association of GA with Body Fat Parameters and Clinical Characteristics

We conducted correlation and partial correlation analysis between anthropometric variables (BMI and W), accurate adiposity variables (%fat, fat mass, FFM, SFA, and VFA), glucose levels, lipid profile, BP, CRP, and GA (Table 3). Accordingly, FPG, HbA1c, and HDL-c were found to be positively correlated with GA (all *P*<0.01). In contrast, all body fat parameters (BMI, W, %fat, fat mass, FFM, SFA, and VFA), as well as TG, LDL-c, and CRP, were found to be negatively correlated with GA (all *P*<0.01). After adjustment for age, BMI, ALB, smoking, anti-hypertensive therapy, and lipid-lowering therapy, the negative correlation between W, %fat, fat mass, SFA, VFA, TG, and GA remained.

To determine which variables were independently associated with GA, multiple stepwise regression analysis was performed (Table 4). The dependent variable was GA, while age, glucose profile, all body fat parameters (BMI, W, %fat, fat mass, FFM, SFA, and VFA), ALB, SBP, DBP, TG, HDL-c, LDL-c, and CRP, as well as smoking status, anti-hypertensive therapy, and lipid-lowering therapy were tested as independent variables. Three regression models were constructed according to the various selected body fat parameters in men, premenopausal women, and postmenopausal women respectively. In model 1, only anthropometric variables (BMI and W) were used, with no accurate adipose variables. Results showed that, in addition to age, FPG, 2hPG, ALB and TG, both BMI and W were independently associated with serum GA. In men, SBP and smoking were also independently associated with serum GA. Model 2 included anthropometric variables (BMI and W) and accurate adiposity variables (%fat, SFA, and VFA). We found that %fat and VFA were two obesity-related factors independently affected GA. In model 3, we replaced %fat with fat mass, and FFM. Results from this model showed that fat mass and VFA were independently correlated with serum GA.

## Discussion

The present study represents the first of its kind to investigate the relation between accurate adiposity variables and GA in a Chinese population. Our results indicated that not only BMI, but also body fat mass and visceral adipose tissue, were independently negatively correlated with GA. Furthermore, subjects with elevated body fat content showed lower GA, regardless of BMI. Additionally, the results suggested that the inverse influence of obesity on GA might be largely attributable to the effects of fat mass and visceral adipose tissue.

Glycated serum proteins (GSP) are formed by a nonenzymatic oxidation reaction that occurs upon binding of blood glucose with plasma proteins, which are composed of 70% ALB. Measurement of GSP is strongly influenced by concentrations of proteins and the exact half-lives of all glycated proteins have not been determined [19]. GA (%) represents the calculated results of percentage of aGA (g/dL) in total ALB (g/dL). GA determined by the present enzymatic method is accurate regardless of the ALB concentration [20]. GA is now widely regarded as a sufficiently accurate reflection of short-term hyperglycemia control (two-week periods) because ALB has a circulating half-life of about 17 days [21]. GA has also been proven useful for distinguishing stress hyperglycemia, and for screening and diagnosing diabetes [22], [23]. Moreover, GA has been reported as a better indicator of glycemic control than HbA1c in hemodialysis patients with end-stage diabetic nephropathy [24].

Unfortunately, perturbations in ALB turnover can affect the accuracy of GA’s indication of glycemic control, as increased turnover of ALB results in lower GA in relation to glycemia; conversely, GA may be higher in conditions of decreased ALB turnover [2]. In addition, ALB level is known to influence its own catabolism in such a way that lower serum concentrations of ALB are catabolized substantially more slowly and vice versa [25]. In our study, we found that ALB was independently negatively correlated with GA, which was in accordance with previous studies [6], [25].

Recent studies have revealed that obesity is also an important factor affecting GA. It is well known that body fat distribution is distinctive between the male and female sexes. In general, women have a higher percentage of body fat than men, and men are more prone to abdominal and visceral obesity [26], [27]. Menopause also influences body fat distribution in women [28], [29]. Therefore, the data analysis in the present study was carried out with stratification among men, premenopausal women, and postmenopausal women. This approach confirmed that both premenopausal and postmenopausal women had significantly higher %fat and fat mass than men, while men had higher VFA. These findings agreed with data from previous reports [26], [27].

In this study, only NGT individuals were selected for study, because in patients with hyperglycemia there was influence on GA due to recent fluctuations of plasma glucose [15]. In addition, the study design included MRI measurement of abdominal obesity, rather than the simple anthropometric index of W that is commonly used in clinic, as the International Diabetes Foundation recommended MRI to be one of “platinum standard” definition of measuring abdominal fat accumulation [30], [31].

Our study showed that %fat, fat mass, and VFA were negatively correlated with serum GA, even after adjusting for age, smoking, BMI, ALB, and current therapies. In the same BMI category, men and postmenopausal women with increased %fat had significantly lower GA than those with lower %fat, which supported the theory that body fat may play a more important role than BMI in affecting GA. While in premenopausal women, the differences did not reach statistical significance (*P* = 0.076 and *P* = 0.087 respectively), which is possibly due to the relatively small sample size of premenopausal women, especially of the non-overweight/non-obese subjects with elevated %fat (n = 18) and the overweight/obese subjects with normal %fat (n = 31). Multiple stepwise regression of all the three groups showed that %fat, fat mass, and VFA were independent explanatory variables for serum GA. Therefore, we speculate that the negative effect of BMI on serum GA observed in our study was mainly due to body fat mass, but not FFM, while visceral adipose tissue also played an important role.

Previous studies have indicated that obesity is negatively associated with GA [4]–[7]. However, the underlying mechanisms of this relation remain unknown. It has been demonstrated that ALB concentrations are low in obese subjects, as compared to their non-obese counterparts, and are negatively correlated with BMI [32]. But, Nishimura et al indicated that obese children had higher serum ALB than non-obese children, and Koga et al. found no correlation between BMI and ALB concentrations [5], [7]. We failed to find significant association between body fat parameters and ALB in men, but only detected a negative correlation between BMI, %fat, fat mass and ALB in premenopausal and postmenopausal women (data not shown). Moreover, the negative association of %fat, fat mass, and VFA with GA remained after adjusting for ALB. Furthermore, we found that body fat parameters, including BMI, W, %fat, fat mass, FFM, SFA, and VFA, were negatively correlated with aGA (data not shown). According to the above results, it is unlikely that the negative association of obesity with GA is due to abnormal ALB concentrations in obese subjects, but may mainly be attributable to changes in aGA.

Inflammation can increase the catabolic rate of albumin and reduce the rate of albumin synthesis [33]. Other than BMI, Koga et al. also observed a significantly inverse correlation between CRP and GA, and subsequently conducted multivariate regression analysis which revealed the independent association of CRP with GA. Thus, the authors of that study hypothesized that one mechanism for increased turnover of serum albumin in obese subjects may be chronic inflammation represented by increased CRP, which would result in lower serum GA in relation to glucose level [7]. Furthermore, they found that smoking, which is known to elevate levels of inflammatory cytokines (e.g. CRP), was negatively associated with serum GA [34], providing further support for this hypothesis.

In our study, we also found that overweight/obese subjects had significantly higher CRP than their non-overweight/non-obese counterparts, and that CRP were negatively correlated with GA. However, CRP did not enter the equation in the multiple regression analysis. It is possible that these findings reflect the fact that the majority of our subjects were healthy people with relatively low CRP. In addition, we cannot rule out the possibility that some other inflammatory cytokines not measured in our study significantly impact GA. As such, our data were unable to determine whether obesity-related chronic inflammation is a primary mechanism for negative influence of obesity on GA.

Besides %fat, fat mass, VFA, FPG, 2hPG, and ALB, we also determined that age was positively associated with serum GA, while TG was negatively associated, in all subjects. In NGT men, in particular, smoking was positively correlated with GA, while SBP was negatively correlated. These results are consistent with previous studies that have reported age, smoking, and TG as independent influencing factors of GA [7], [34], [35]. In order to indentify whether any of the variables mainly exerted an influence on ALB or aGA, we conducted correlation analysis (data not shown), and found that age mainly affected ALB, while smoking, SBP, and TG had a more predominant influence on aGA. The mechanism underlying these effects, however, remains unknown.

There were two limitations to the present study that must be considered when interpreting the results. First, the size of the study population was not large enough, especially for the obesity subjects. Second, the cross-sectional study design precluded observations of future variations.

In conclusion, when monitoring hyperglycemic control by GA the actual plasma glucose levels may be underestimated in obese patients or patients with central obesity. In patients who have appropriate BMI but elevated body fat content, GA may also be lower than the actual plasma glucose levels. Although the potential mechanism remains unknown, the effects of obesity and body fat on GA merit consideration when using GA as an indicator of glycemic control in clinic.
